# The Prevalence of Chronic Medical Diseases in Individuals With Psychiatric Disorders: A Retrospective Study in Saudi Arabia

**DOI:** 10.7759/cureus.82368

**Published:** 2025-04-16

**Authors:** Moayyad Alsalem, Ahmad M Alsaleh, Abdulmjeed Alamri, Mohammed Bogari, Basil Alghamdi, Alwaleed K Aloufi, Abdulelah Niaz

**Affiliations:** 1 Internal Medicine, Psychiatry Section, King Saud Bin Abdulaziz University for Health Sciences, Jeddah, SAU; 2 Psychiatry, King Abdulaziz Medical City, Jeddah, SAU; 3 Psychiatry, King Saud Bin Abdulaziz University for Health Sciences, Jeddah, SAU; 4 College of Medicine, King Saud Bin Abdulaziz University for Health Sciences, Jeddah, SAU

**Keywords:** chronic diseases, comorbidity, mental health, non-communicable diseases, psychiatric disorders

## Abstract

Background

Psychiatric disorders are conditions that affect thinking, behavior, and overall mental health, influencing daily functioning and quality of life. Chronic diseases are persistent health conditions that necessitate ongoing medical treatment and management. This study aims to assess the prevalence of chronic medical diseases among individuals with psychiatric disorders in Saudi Arabia.

Materials and methods

A retrospective cohort study was conducted at King Abdulaziz Medical City, Jeddah, utilizing electronic health records from May 2016 to December 2024. The study included psychiatric patients aged ≥17 years. A minimal sample size of 296 was determined using Raosoft software (Raosoft Inc., Seattle, Washington, United States), assuming the prevalence of 50%, a 95% confidence level, and a 5% margin of error. Data were collected using a random sampling technique and categorized into three sections: demographic characteristics, psychiatric diagnoses and treatments, and chronic disease status. Pearson’s Chi-squared test and Fisher’s exact test were used to assess associations between psychiatric disorders and chronic diseases.

Results

A total of 361 psychiatric patients (median age of 54 years) were included, with 222 (61%) patients being female and 272 (77%) being unemployed. A total of 202 patients (56%) were diagnosed with major depressive disorder (MDD), 81 (22%) were diagnosed with generalized anxiety disorder (GAD), and 32 (8.9%) had bipolar disorder; 290 psychiatric patients (80.33%) had a chronic disease and 188 patients (52.1%) used selective serotonin reuptake inhibitors (SSRIs). MDD with psychotic symptoms (p=0.025), conversion disorder (p=0.038), and post-traumatic stress disorder (PTSD) (p<0.0001) were not associated with chronic diseases. Bipolar disorder and adjustment disorder were significantly associated with cancer (p=0.033, p=0.006). PTSD was linked to hypertension (p=0.033) and diabetes (p=0.014), while delirium was significantly associated with ischemic heart disease (p<0.001) and stroke (p=0.004).

Conclusion

The high prevalence of chronic diseases among psychiatric patients emphasizes the need for integrated healthcare approaches to manage comorbid conditions effectively. Addressing mental and physical health simultaneously may improve patient outcomes and quality of life.

## Introduction

Psychiatric disorders are conditions that interfere with the ability to manage emotions, cognitive processes, and behavior, affecting mental well-being and daily life. These conditions can lead to considerable distress and impair an individual's ability to fulfil personal, social, and family obligations. In the United States, it is estimated that over 20% of adults are affected by psychiatric disorders [1]. Similarly, a study from Saudi Arabia indicated that 28.5% of patients visiting primary healthcare centers reported experiencing a psychiatric disorder [2].

Noncommunicable diseases (NCDs), also known as chronic diseases, tend to be of long duration and are the result of a combination of genetic, physiological, environmental, and behavioral factors. These diseases often interfere with daily functioning and require continuous medical management. Such conditions can severely impact patients' quality of life and are associated with serious complications, including increased mortality. Chronic diseases include various conditions such as cardiovascular diseases, diabetes, cancer, and chronic respiratory disorders [3].

In 2017, these diseases accounted for 73.4% of global deaths, reflecting a 22.7% increase since 2007, largely attributed to cardiovascular diseases (CVDs) [4,5]. NCDs contribute to 35% of all deaths in Saudi Arabia, where CVDs lead to 28% of NCD-related deaths, followed by cancer, diabetes, and chronic respiratory conditions [6]. This rising trend in NCDs has been associated with an increase in disability and a deterioration in the quality of life within Saudi Arabia [7].

Research indicates a significant correlation between psychiatric disorders and chronic diseases, where individuals with one condition are more likely to experience the other. This co-occurrence can complicate diagnosis and treatment, exacerbating the overall health burden. A systematic review and meta-analysis found that individuals with multimorbidity are two to three times more likely to develop depression compared to those without multiple chronic conditions [8]. Additionally, psychiatric patients have a 27% higher risk of developing chronic diseases and are 31% more likely to experience multimorbidity [9]. Among psychiatric inpatients, chronic conditions such as neurological disorders, liver diseases, and chronic pulmonary conditions are prevalent, although the type of chronic disease may vary depending on the psychiatric diagnosis. For example, liver disease is commonly observed in patients with mood disorders, whereas neurological disorders are more prevalent among those with schizophrenia-related conditions [10]. Individuals with severe mental illnesses, including schizophrenia, bipolar disorder, and major depressive disorder, exhibit a pooled prevalence of CVD at 9.9% (95%CI: 7.4-13.3) [11]. A study conducted in Sweden reported that within five years of diagnosis, 25-32% of patients with chronic conditions such as respiratory diseases, CVDs, or diabetes had a co-occurring lifetime diagnosis of a psychiatric disorder [12].

The presence of psychiatric disorders can contribute to both the development and progression of chronic medical illnesses. This may result from factors such as inadequate self-care, adverse effects of psychiatric medications, and the physiological impact of mental health symptoms, including stress and depression, which can weaken the immune system, promote inflammation, and lead to unhealthy lifestyle habits. For example, anxiety and depression are common among patients with chronic pain, often intensifying the perception of pain and complicating treatment strategies [13]. Therefore, psychiatric patients face an elevated risk for various chronic diseases, underscoring the need for integrated healthcare strategies. This study aims to assess the overall prevalence of chronic diseases in individuals with psychiatric disorders.

## Materials and methods

Study design and setting

This retrospective cohort study was conducted in Saudi Arabi at King Abdulaziz Medical City in Jeddah, a tertiary healthcare facility with a psychiatric department. The hospital’s comprehensive medical and psychiatric services make it an ideal setting for examining the prevalence of chronic diseases in individuals with psychiatric disorders. The study design was chosen to leverage existing electronic health records for a comprehensive assessment of psychiatric disorders and their associations with chronic diseases over an extended period.

Study population

The study population included all psychiatric patients aged 17 years and above diagnosed based on clinicians' assessments and the Diagnostic and Statistical Manual of Mental Disorders, Fifth Edition (DSM-5) criteria between May 2016 and December 2024. Patients younger than 17 years of age were excluded due to the lower prevalence of chronic diseases in younger age. Additionally, patients with missing or incomplete medical records were excluded to ensure data integrity.

****Sample size calculation

The required sample size was calculated using the Raosoft sample size calculator (Raosoft Inc., Seattle, Washington, United States). Given the total psychiatric patient population of 1200 between 2016 and 2024, we assumed a prevalence of 50% (as a conservative estimate in the absence of prior local prevalence data) with a 95% confidence level and a 5% margin of error. The time period was chosen based on the fact that there were no data prior to this date on the hospital system. The minimum sample size required was calculated to be 296 patients. Although the minimum calculated sample size was 296, a total of 361 patients were included to increase the study's statistical power and improve the generalizability of the findings.

Sampling technique and data collection

From a population of 1200 patients, a sample of 361 was selected using simple random sampling via a random number generator (https://www.randomizer.org), minimizing potential selection bias. Data collection was carried out by a group of trained medical students, who extracted data from the BestCare system (ezCaretech Co., Ltd, Seoul, South Korea), a hospital-wide electronic medical record (EMR) system that ensures standardized documentation. All data collectors underwent standardized training to ensure uniformity in data extraction and to reduce inter-observer variability. To ensure reliability, 10% of the selected records were independently reviewed by a senior researcher. Any data entry discrepancies identified during this review were resolved through discussion, during which the original medical records were re-examined collaboratively to reach a consensus ensuring data accuracy and consistency. The data were extracted 

The collected data was divided into three sections: (i) the first was demographic characteristics of the patients, including gender, age at the diagnosis, marital status, and occupation, (ii) the second dealt with the type of psychiatric disorder, prescribed medication, therapy/counseling, and hospitalization, and (iii) the third evaluated the state of chronic disease of the patients including hypertension, diabetes mellitus, ischemic heart disease, stroke, peripheral vascular disease, obstructive sleep apnea, osteoarthritis, cancer, autoimmune thyroid disease, celiac disease, retinopathy, nephropathy, and neuropathy.

Statistical analysis

Data cleaning and preprocessing were performed in Microsoft Excel (Microsoft Corporation, Redmond, Washington, United States) before importing into IBM SPSS Statistics for Windows, Version 27.0 (Released 2020; IBM Corp., Armonk, New York, United States) for analysis. Continuous variables were assessed for normality using the Kolmogorov-Smirnov test and visual inspection of histograms. Categorical variables were presented as frequencies and percentages, while non-normally distributed continuous variables were summarized using the median and interquartile range (IQR). Pearson’s Chi-squared test and Fisher’s exact test were used to assess associations between psychiatric disorders and chronic diseases. A p-value of <0.05 was considered statistically significant.

Ethical considerations

Ethical approval was obtained from the Institutional Review Board of King Abdullah International Medical Research Center (KAIMRC) (approval number: IRB/0828/24). The study adhered to the Declaration of Helsinki ethical guidelines. Given the retrospective nature of the study, the requirement for informed consent was waived by the IRB. Patient confidentiality was rigorously maintained, with data anonymized and stored securely. To ensure anonymity and confidentiality, patient data were collected from medical records without including personal identifiers such as names or hospital ID numbers.

## Results

A total of 1200 records were reviewed, and 361 records of psychiatric patients were included with a median age of 54 years at diagnosis. Of these, 222 patients (61%) were female, 274 (77%) were married, 272 (77%) were unemployed, 44 (12%) were employed, and 37 (10%) were retired (Table 1).

**Table 1:** Demographic characteristics of study participants (N=361)

Characteristics	Frequency (Percentage)
Sex	
Female	222 (61%)
Male	139 (39%)
Age at diagnosis of psychiatric disease, median (IQR)	54 (38-66)
Material status	
Divorced	20 (5.6%)
Married	274 (77%)
Single	64 (18%)
Missing data	3
Occupation	
Employed	44 (12%)
Retired	37 (10%)
Unemployed	272 (77%)
Missing data	8

Among the patients, 202 (56%) were diagnosed with MDD, 81 (22%) were diagnosed with generalized anxiety disorder (GAD), 32 (8.9%) had bipolar disorder, 27 (7.5%) had adjustment disorder, and 22 (6.1%) were diagnosed with schizophrenia. Only 75 (21%) got therapy/consulting, and only 24 (6.6%) were hospitalized(Table 2).

**Table 2:** Characteristics related to psychiatric diagnosis (N=361) ^*^Multiple selections were possible for this question MMD: major depressive disorder; GAD: generalized anxiety disorder; PTSD: post-traumatic stress disorder

Characteristic	Frequency (Percentage)
Psychiatric disorder*	
MDD	202 (56%)
GAD	81 (22%)
Bipolar Disorder	32 (8.9%)
Adjustment Disorder	27 (7.5%)
Schizophrenia	22 (6.1%)
Delirium	16 (4.4%)
PTSD	6 (1.7%)
Unspecified psychotic disorder	5 (1.4%)
unspecified anxiety disorder	4 (1.1%)
MDD with psychotic symptoms	4 (1.1%)
Substance use disorder	3 (0.8%)
Conversion Disorder	2 (0.6%)
MDD with anxious distress	1 (0.3%)
Substance-induced Psychosis	1 (0.3%)
Insomnia	1 (0.3%)
Factitious Disorder	1 (0.3%)
Cluster B Personality Traits	1 (0.3%)
Social Phobia	1 (0.3%)
Panic disorder	1 (0.3%)
Therapy/Counseling	
No	283 (79%)
Yes	75 (21%)
Missing	3
Hospitalizations	
No	337 (93%)
Yes	24 (6.6%)

**Figure 1:**
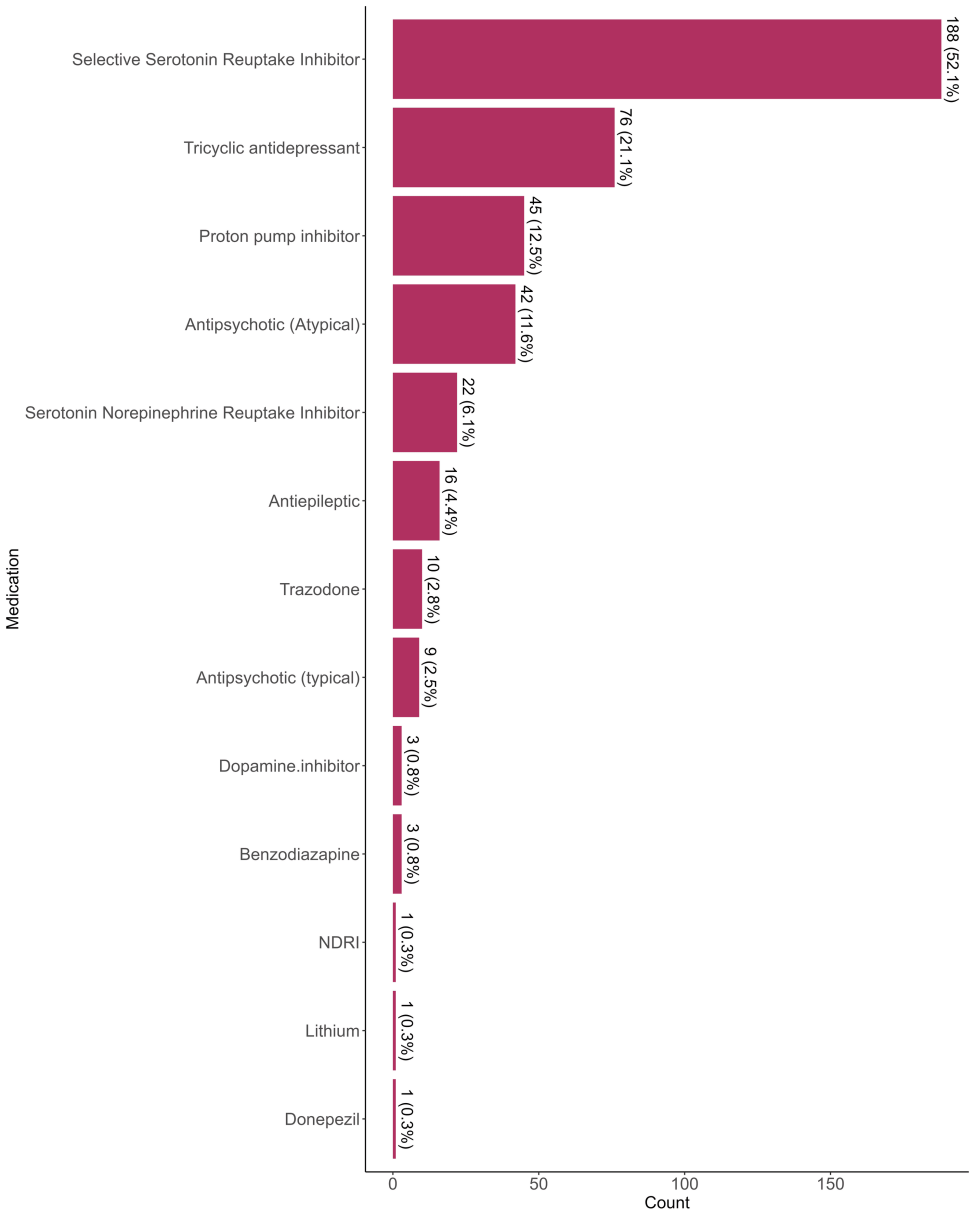
Prescribed medication for psychiatric patients NDRI: norepinephrine-dopamine reuptake inhibitor

The most commonly used medication category was selective serotonin reuptake inhibitors (SSRIs), prescribed for 188 patients (52.1%). Tricyclic antidepressants (TCAs) were prescribed for 76 patients (21.1%), proton pump inhibitors (PPIs) for 45 patients (12.5%), and atypical antipsychotics were prescribed for 42 patients (11.6%). Serotonin-norepinephrine reuptake inhibitors (SNRIs) were given in 22 patients (6.1%). Antiepileptic drugs were used in 16 patients (4.4%), while trazodone was used in 10 patients (2.8%). Typical antipsychotics were prescribed to nine patients (2.5%). Dopamine inhibitors and benzodiazepines were each used for three patients (0.8%). Less commonly, norepinephrine-dopamine reuptake inhibitors (NDRIs), lithium, and donepezil had been used in one patient each (0.3%) (Figure 1).

A total of 290 (80.33%) had a chronic disease. Patients diagnosed with MDD, with psychotic symptoms (p=0.025), conversion disorder (p=0.038), or PTSD (p< 0.0001) were associated with having no chronic diseases(Table 3).

**Table 3:** Association between psychiatric disorder and chronic diseases ^2^Pearson's Chi-squared test; Fisher's exact test MDD: ; GAD: generalized anxiety disorder; PTSD: post-traumatic stress disorder

Psychiatric disorder	No chronic disease (n = 71), n (%)	Presence of chronic disease (n = 290), n (%)	p-value^2^
Generalized anxiety disorder			0.3
No	52 (73%)	228 (79%)	-
Yes	19 (27%)	62 (21%)	-
Panic disorder			>0.9
No	71 (100%)	289 (100%)	-
Yes	0 (0%)	1 (0.3%)	-
Major depressive disorder			0.5
No	34 (48%)	125 (43%)	-
Yes	37 (52%)	165 (57%)	-
Bipolar Disorder			0.4
No	63 (89%)	266 (92%)	-
Yes	8 (11%)	24 (8.3%)	-
Schizophrenia			0.8
No	66 (93%)	273 (94%)	-
Yes	5 (7.0%)	17 (5.9%)	-
Adjustment Disorder			0.5
No	67 (94%)	267 (92%)	-
Yes	4 (5.6%)	23 (7.9%)	-
Social Phobia			>0.9
No	71 (100%)	289 (100%)	-
Yes	0 (0%)	1 (0.3%)	-
MDD with psychotic symptoms			0.025
No	68 (96%)	289 (100%)	-
Yes	3 (4.2%)	1 (0.3%)	-
Substance use disorder			>0.9
No	71 (100%)	287 (99%)	-
Yes	0 (0%)	3 (1.0%)	-
MDD with anxious distress			0.2
No	70 (99%)	290 (100%)	-
Yes	1 (1.4%)	0 (0%)	-
Substance-induced Psychosis			0.2
No	70 (99%)	290 (100%)	-
Yes	1 (1.4%)	0 (0%)	-
Conversion Disorder			0.038
No	69 (97%)	290 (100%)	-
Yes	2 (2.8%)	0 (0%)	-
Unspecified psychotic disorder			>0.9
No	70 (99%)	286 (99%)	-
Yes	1 (1.4%)	4 (1.4%)	-
Insomnia			>0.9
No	71 (100%)	289 (100%)	-
Yes	0 (0%)	1 (0.3%)	-
Factitious Disorder			>0.9
No	71 (100%)	289 (100%)	-
Yes	0 (0%)	1 (0.3%)	-
Unspecified anxiety disorder			>0.9
No	71 (100%)	286 (99%)	-
Yes	0 (0%)	4 (1.4%)	-
PTSD			<0.001
No	65 (92%)	290 (100%)	-
Yes	6 (8.5%)	0 (0%)	-
Cluster B Personality Traits			0.2
No	70 (99%)	290 (100%)	-
Yes	1 (1.4%)	0 (0%)	-
Delirium			0.7
No	69 (97%)	276 (95%)	-
Yes	2 (2.8%)	14 (4.8%)	-

Individuals with cancer showed a higher prevalence of bipolar disorder compared to those without cancer (p = 0.033). Similarly, adjustment disorder demonstrated a significant association with cancer (p = 0.006), where 87% of those with cancer did not have adjustment disorder, compared to 95% in the non-cancer group. For PTSD, there was a significant association with both hypertension (p = 0.033) and diabetes mellitus (p = 0.014). All patients with diabetes and hypertension who were categorized as having PTSD did not exhibit symptoms of the disorder. Delirium showed significant associations with hypertension (p = 0.004), diabetes mellitus (p = 0.044), ischemic heart disease (p < 0.001), and stroke (p = 0.004). The prevalence of delirium was notably higher among individuals with these medical conditions (Table 4).

**Table 4:** Association between chronic illness and psychiatric disorders MDD: major depressive disorder; GAD: generalized anxiety disorder; PTSD: post-traumatic stress disorder ^2^Pearson's Chi-squared test; Fisher's exact test

Characteristic	Hypertension, n (%)			Diabetes mellitus, n (%)			Ischemic heart disease, n (%)			Stroke, n (%)			Cancer, n (%)		
	No (n=195)	Yes (n=166)	p-value^2^	No (n=179)	Yes (n=182)	p-value^2^	No (n=300)	Yes (n=61)	p-value^2^	No (n=334)	Yes (n=27)	p-value^2^	No (n=257)	Yes (n=104)	p-value^2^
GAD			0.11			0.5			0.2			>0.9			0.14
No	145 (74%)	135 (81%)		136 (76%)	144 (79%)		229 (76%)	51 (84%)		259 (78%)	21 (78%)		194 (75%)	86 (83%)	
Yes	50 (26%)	31 (19%)		43 (24%)	38 (21%)		71 (24%)	10 (16%)		75 (22%)	6 (22%)		63 (25%)	18 (17%)	
Panic disorder			>0.9			0.5			>0.9			>0.9			0.3
No	194 (99%)	166 (100%)		178 (99%)	182 (100%)		299 (100%)	61 (100%)		333 (100%)	27 (100%)		257 (100%)	103 (99%)	
Yes	1 (0.5%)	0 (0%)		1 (0.6%)	0 (0%)		1 (0.3%)	0 (0%)		1 (0.3%)	0 (0%)		0 (0%)	1 (1.0%)	
MDD			0.8			>0.9			>0.9			0.7			0.5
No	87 (45%)	72 (43%)		79 (44%)	80 (44%)		132 (44%)	27 (44%)		148 (44%)	11 (41%)		116 (45%)	43 (41%)	
Yes	108 (55%)	94 (57%)		100 (56%)	102 (56%)		168 (56%)	34 (56%)		186 (56%)	16 (59%)		141 (55%)	61 (59%)	
Bipolar Disorder			0.5			0.2			0.8			0.5			0.033
No	176 (90%)	153 (92%)		167 (93%)	162 (89%)		273 (91%)	56 (92%)		303 (91%)	26 (96%)		229 (89%)	100 (96%)	
Yes	19 (9.7%)	13 (7.8%)		12 (6.7%)	20 (11%)		27 (9.0%)	5 (8.2%)		31 (9.3%)	1 (3.7%)		28 (11%)	4 (3.8%)	
Schizophrenia			0.6			0.2			>0.9			0.7			0.5
No	182 (93%)	157 (95%)		165 (92%)	174 (96%)		281 (94%)	58 (95%)		314 (94%)	25 (93%)		240 (93%)	99 (95%)	
Yes	13 (6.7%)	9 (5.4%)		14 (7.8%)	8 (4.4%)		19 (6.3%)	3 (4.9%)		20 (6.0%)	2 (7.4%)		17 (6.6%)	5 (4.8%)	
Adjustment Disorder			0.8			0.15			>0.9			>0.9			0.006
No	181 (93%)	153 (92%)		162 (91%)	172 (95%)		277 (92%)	57 (93%)		309 (93%)	25 (93%)		244 (95%)	90 (87%)	
Yes	14 (7.2%)	13 (7.8%)		17 (9.5%)	10 (5.5%)		23 (7.7%)	4 (6.6%)		25 (7.5%)	2 (7.4%)		13 (5.1%)	14 (13%)	
Social Phobia			>0.9			0.5			>0.9			>0.9			0.3
No	194 (99%)	166 (100%)		178 (99%)	182 (100%)		299 (100%)	61 (100%)		333 (100%)	27 (100%)		257 (100%)	103 (99%)	
Yes	1 (0.5%)	0 (0%)		1 (0.6%)	0 (0%)		1 (0.3%)	0 (0%)		1 (0.3%)	0 (0%)		0 (0%)	1 (1.0%)	
MDD with psychotic symptoms			0.13			0.4			>0.9			>0.9			0.6
No	191 (98%)	166 (100%)		176 (98%)	181 (99%)		296 (99%)	61 (100%)		330 (99%)	27 (100%)		253 (98%)	104 (100%)	
Yes	4 (2.1%)	0 (0%)		3 (1.7%)	1 (0.5%)		4 (1.3%)	0 (0%)		4 (1.2%)	0 (0%)		4 (1.6%)	0 (0%)	
Substance use disorder			0.3			0.12			>0.9			>0.9			>0.9
No	192 (98%)	166 (100%)		176 (98%)	182 (100%)		297 (99%)	61 (100%)		331 (99%)	27 (100%)		255 (99%)	103 (99%)	
Yes	3 (1.5%)	0 (0%)		3 (1.7%)	0 (0%)		3 (1.0%)	0 (0%)		3 (0.9%)	0 (0%)		2 (0.8%)	1 (1.0%)	
MDD with anxious distress			>0.9			0.5			>0.9			>0.9			>0.9
No	194 (99%)	166 (100%)		178 (99%)	182 (100%)		299 (100%)	61 (100%)		333 (100%)	27 (100%)		256 (100%)	104 (100%)	
Yes	1 (0.5%)	0 (0%)		1 (0.6%)	0 (0%)		1 (0.3%)	0 (0%)		1 (0.3%)	0 (0%)		1 (0.4%)	0 (0%)	
Substance induced Psychosis			>0.9			0.5			>0.9			>0.9			>0.9
No	194 (99%)	166 (100%)		178 (99%)	182 (100%)		299 (100%)	61 (100%)		333 (100%)	27 (100%)		256 (100%)	104 (100%)	
Yes	1 (0.5%)	0 (0%)		1 (0.6%)	0 (0%)		1 (0.3%)	0 (0%)		1 (0.3%)	0 (0%)		1 (0.4%)	0 (0%)	
Conversion Disorder			0.5			0.2			>0.9			>0.9			>0.9
No	193 (99%)	166 (100%)		177 (99%)	182 (100%)		298 (99%)	61 (100%)		332 (99%)	27 (100%)		255 (99%)	104 (100%)	
Yes	2 (1.0%)	0 (0%)		2 (1.1%)	0 (0%)		2 (0.7%)	0 (0%)		2 (0.6%)	0 (0%)		2 (0.8%)	0 (0%)	
Unspecified psychotic disorder			0.4			0.2			0.6			>0.9			0.6
No	191 (98%)	165 (99%)		175 (98%)	181 (99%)		295 (98%)	61 (100%)		329 (99%)	27 (100%)		254 (99%)	102 (98%)	
Yes	4 (2.1%)	1 (0.6%)		4 (2.2%)	1 (0.5%)		5 (1.7%)	0 (0%)		5 (1.5%)	0 (0%)		3 (1.2%)	2 (1.9%)	
Insomnia			0.5			0.5			>0.9			>0.9			0.3
No	195 (100%)	165 (99%)		178 (99%)	182 (100%)		299 (100%)	61 (100%)		333 (100%)	27 (100%)		257 (100%)	103 (99%)	
Yes	0 (0%)	1 (0.6%)		1 (0.6%)	0 (0%)		1 (0.3%)	0 (0%)		1 (0.3%)	0 (0%)		0 (0%)	1 (1.0%)	
Factitious Disorder			>0.9			>0.9			>0.9			>0.9			>0.9
No	194 (99%)	166 (100%)		179 (100%)	181 (99%)		299 (100%)	61 (100%)		333 (100%)	27 (100%)		256 (100%)	104 (100%)	
Yes	1 (0.5%)	0 (0%)		0 (0%)	1 (0.5%)		1 (0.3%)	0 (0%)		1 (0.3%)	0 (0%)		1 (0.4%)	0 (0%)	
Unspecified anxiety disorder			0.3			0.6			0.5			>0.9			>0.9
No	194 (99%)	163 (98%)		178 (99%)	179 (98%)		297 (99%)	60 (98%)		330 (99%)	27 (100%)		254 (99%)	103 (99%)	
Yes	1 (0.5%)	3 (1.8%)		1 (0.6%)	3 (1.6%)		3 (1.0%)	1 (1.6%)		4 (1.2%)	0 (0%)		3 (1.2%)	1 (1.0%)	
PTSD			0.033			0.014			0.6			>0.9			0.2
No	189 (97%)	166 (100%)		173 (97%)	182 (100%)		294 (98%)	61 (100%)		328 (98%)	27 (100%)		251 (98%)	104 (100%)	
Yes	6 (3.1%)	0 (0%)		6 (3.4%)	0 (0%)		6 (2.0%)	0 (0%)		6 (1.8%)	0 (0%)		6 (2.3%)	0 (0%)	
Cluster B Personality Traits			>0.9			0.5			>0.9			>0.9	-		>0.9
No	194 (99%)	166 (100%)		178 (99%)	182 (100%)		299 (100%)	61 (100%)		333 (100%)	27 (100%)		256 (100%)	104 (100%)	
Yes	1 (0.5%)	0 (0%)		1 (0.6%)	0 (0%)		1 (0.3%)	0 (0%)		1 (0.3%)	0 (0%)		1 (0.4%)	0 (0%)	
Delirium			0.004			0.044			<0.001			0.004			>0.9
No	192 (98%)	153 (92%)		175 (98%)	170 (93%)		293 (98%)	52 (85%)		323 (97%)	22 (81%)		245 (95%)	100 (96%)	
Yes	3 (1.5%)	13 (7.8%)		4 (2.2%)	12 (6.6%)		7 (2.3%)	9 (15%)		11 (3.3%)	5 (19%)		12 (4.7%)	4 (3.8%)	

## Discussion

This study aimed to assess the prevalence of different psychiatric illnesses among patients with chronic diseases. The majority were female patients with a median age of 54 at diagnosis. This could be due to the fact that women, overall, might express more psychological distress with chronic diseases.

Participants were diagnosed with a variety of psychiatric illnesses; the majority had MDD (56%), followed by GAD (22%). A much smaller number of participants were diagnosed with bipolar disorder (8.9%), adjustment disorder (7.5%), and schizophrenia (6.1%). Among all these patients with different psychiatric illnesses, only 21% got therapy/consulting, and only 6.6% were hospitalized. The most commonly prescribed medication was SSRIs (52%), followed by tricyclic antidepressants (21%), and atypical antipsychotics (11%). Apart from psychiatric medications, PPIs were prescribed for about 12% of patients, and antiepileptic drugs constituted 4.43% of the prescriptions. PPIs could be prescribed for primary diseases or as a cover for other chronic medications that have GI side effects.

The majority (n=290, 80%) with psychiatric illnesses were also diagnosed with chronic diseases. Individuals with cancer showed a higher prevalence of bipolar and adjustment disorders. On the other hand, delirium was more prevalent among patients with hypertension, diabetes mellitus, ischemic heart diseases, and stroke. Patients with chronic physical illness have more admissions due to psychiatric illnesses (like MDD) compared to those without physical illnesses [14]. A review article showed that there is a strong association between chronic illness and psychiatric illnesses among adolescents [15]. For example, there were more prevalent anxiety and depressive disorders among adolescents with asthma. Adolescent cancer survivors also showed a high prevalence of post-traumatic stress disorders, clinical depression, and anxiety [16].

Cancer patients with psychiatric disorders have impaired adherence to medications and lower quality of life. A study reported a high incidence of psychiatric illnesses among cancer patients of up to 97%; these included adjustment, mood, and anxiety disorders [17]. Risk factors for such high incidence of psychiatric illnesses include cancer recurrence, poor social support, low income, and other chronic diseases [17,18]. This indicates that the issue of psychological stress and psychiatric illness among chronic disease patients is a huge burden across varying age groups and physical illness.

In patients with diabetes (type 1 and 2), 42% were diagnosed with at least one psychiatric disease, with GAD being the most common, followed by dysthymia, social phobia, and panic disorder [19]. Around 2% of patients experienced suicidal risks. The diagnosis of a chronic medical condition often necessitates significant lifestyle and psychological adjustments, which can increase the vulnerability to psychiatric disorders. Overall, chronically ill patients are estimated to have a two-fold increase in psychiatric illness risks compared to the general population [20,21]. The relationship could also be on the opposite side, where psychiatric illness (especially depressive disorders) are associated with precipitating chronic diseases, including type 2 diabetes and cardiovascular disease. Depression can additionally impact the course and outcome of these diseases [22]. Therefore, prompt identification and effective management of psychological distress in individuals with chronic illnesses are vital components of comprehensive healthcare [23].

Limitations

This study is limited by its lower external validity, which can jeopardize its generalizability. Stigma related to psychiatric disorders could also lead to underrepresentation. Wider population and national studies are recommended. 

## Conclusions

The study revealed prevalent psychiatric illnesses among patients with chronic diseases. The most common of these are MDD, GAD, and bipolar disorders. Cancer patients showed a high prevalence of bipolar disorders, while patients with hypertension and diabetes had more association with PTSD. Patients with chronic diseases exhibit a high psychiatric burden, which can affect medication adherence, treatment outcomes, and quality of life. Hence, this should be taken into consideration when treating chronically ill patients. Healthcare workers should give the psychiatric aspect of patient health more attention to maximize treatment success.
